# Chinese English as a Foreign Language Teachers’ Job Satisfaction, Resilience, and Their Psychological Well-Being

**DOI:** 10.3389/fpsyg.2021.800417

**Published:** 2022-02-09

**Authors:** Wenjing Han

**Affiliations:** School of College English Teaching and Research, Henan University, Kaifeng, China

**Keywords:** job satisfaction, teachers’ well-being, motivation, language teaching, resilience

## Abstract

Job satisfaction, resilience, and teacher well-being, as the three major psychological variables emotioncy- based education, have received special attention among English as a foreign language (EFL) researchers. To pursue the line of this inquiry, this particular study aimed to investigate the relationship between Chinese EFL teachers’ job satisfaction, resilience, and their well-being. To conduct the study, 343 Chinese EFL teachers with different academic qualifications, various academic degrees, and different majors voluntarily participated in this study. The results of the study showed that job satisfaction and resilience could jointly predict 56.4 of the variance in psychological well-being. Both variables were the significant predictors of well-being, while job satisfaction was a better predictor, uniquely explaining 29.6 of well-being’s variance. Based on the findings, some pedagogical implication for administrators, educational institutions, and EFL teachers were discussed in the article.

## Introduction

The foundation of prosperity and self-sufficiency of any society is based on the existence of its educational and research organizations. Most experts and thinkers on educational issues believe that among all the factors affecting the performance of an educational organization, teachers are the most influential factor in the education process (Acton and Glasgow, 2015; Han and Wang, 2021). Teachers can provide a suitable context for attaining better learning by controlling different variables and pave the way for an adequate change in them (Mellati et al., 2013; Lindqvist et al., 2014). However, teaching is considered as a very stressful job and teachers suffer more from mental health problems than other occupations (Richards et al., 2016). Mental health is a state of well-being in which teachers are conscious about their abilities and are able to cope with the natural stresses of life and fulfill their responsibilities in an acceptable way to play their part in a society properly (Buchanan, 2012). Since teachers spend most of their lives in classrooms and educational settings, factors affecting their well-being are undoubtedly related to their job position (Conley and You, 2009; Derakhshan et al., 2021). Furthermore, most studies show that English as a foreign language (EFL) teachers are more susceptible to job-related pressure, mental distress, and burnout than many other occupations (Bowling et al., 2010; Castro et al., 2010; Mellati and Khademi, 2015; Sun and Xia, 2018; Derakhshan et al., 2020; Wang and Guan, 2020). Therefore, an increasing number of studies have been conducted on the factors that influence EFL teachers’ well-being. Among that, many studies reveal that teachers’ job satisfaction (Wang et al., 2015) and resilience (Gu and Day, 2007) are the most important factors which influence EFL teachers’ mental health and well-being. According to the previous studies, teachers, who have a higher level of job satisfaction and resilience, pay more attention to a wide range of job opportunities (Kangas-Dick and O’Shaughnessy, 2020) and have more job success (Puni et al., 2018); they choose higher personal goals (Perera et al., 2018) and have better mental health (Burić and Macuka, 2018; Mellati and Khademi, 2018). In addition, job satisfaction and resilience can increase teachers’ well-being and ability to do things and make them more resilient to job stress (Mousavi et al., 2012; Fathi et al., 2021). Among the most important social issues in teachers’ well-being studies, these two factors have been received special attention in the last two decades (Carmeli et al., 2009).

Despite the enormous research on teachers’ well-being, there is relative scarcity of the research on Chinese EFL teachers’ job satisfaction and resilience. Researchers may neglect the role of Chinese EFL teachers’ job satisfaction in learning to teach and how Chinese EFL teachers’ resilience relates to their well-being. There is a paucity of the research about how Chinese EFL teachers regulate their emotions, the relationship between their emotions and learning. So the present study aims to investigate the relationship among Chinese EFL teachers’ job satisfaction, resilience, and their psychological well-being. This article begins with the empirical literature that focuses on the literature related to teachers’ job satisfaction, resilience, and their psychological well-being. Next is an analysis of an empirical study on the relationship among Chinese EFL teachers’ job satisfaction, resilience, and their psychological well-being. Finally, several future directions for research are suggested.

## Review of Literature

### Teacher Well-Being

Recently, teacher well-being, which is rooted in positive psychology, has received special attention in teacher training programs (Mikus and Teoh, 2021). Recent studies show that people have four different types of well-being: emotional, spiritual, physical, and mental or intellectual. In addition, it is assumed that teacher well-being is inextricably linked to students’ outcomes, progress, motivation, and participation. As a result, full attention to the welfare of the language teacher is essential (Zee and Koomen, 2016).

Research studies show that one of the rationales behind various approaches to teacher well-being is the welfare perspective, which emphasizes purpose, sense of meaning, real-world communication, and the realization of potential abilities (Vesely et al., 2013; Klassen and Durksen, 2014). These studies show that institutions should appreciate the benefits of their organization, teachers, and students to promote teachers’ well-being (Sinclair, 2008; Lee, 2017). They present various characteristics of the factors such as teachers’ job satisfaction and resilience that may influence their well-being (Kokkinos, 2007; Simbula, 2010).

### Job Satisfaction

Job satisfaction as one of the factors of positive psychology is an element that augments competence as well as individual feeling. It can be deduced as an affirmative attitude of EFL teachers toward their educational tasks, teaching settings, and students. Different researchers have presented various definitions for job satisfaction from different perspectives (Capecchi and Piccolo, 2016). Fulfillment and contentment are other terms that are often used interchangeably in the literature for the concept of job satisfaction (Abate et al., 2018; Berliana et al., 2018). Researchers define job satisfaction as a general concept and evaluate a person’s attitude toward the job as a general attitude. In addition to this thinking, some researchers consider job satisfaction as a set of individual attitudes toward different aspects of the job (Kokkinos, 2007; Lent et al., 2011; Mellati et al., 2018). Most experts agree on the relationship between the job and social, and psychological factors. They believe that if a profession offers the preferred pleasure for a teacher, then the teacher is satisfied with his or her profession (Dwiyanti et al., 2019). They consider job satisfaction as a multidimensional and complex concept that is related to physical, psychological, and social factors. Not just one element leads to job satisfaction, but a certain combination of different factors causes a teacher to feel satisfied with his or her job at a specific time (Fathi et al., 2020; Dreer, 2021). An amalgamation of various factors, internal factors, such as the feeling of enjoyment of the work, and external factors, such as work environment, relations, and salaries benefits cause a person to be satisfied with their professions (Escardíbul and Afcha, 2017). Its absence also has numerous effects such as unauthorized absences, reduction of job commitment, complaints, and early retirement that ultimately lead to the poor efficiency and effectiveness of the educational environment (Lee, 2017; Lee and Shin, 2017; Alavi et al., 2021). In principle, a dissatisfied teacher cannot train a learner due to low motivation, high violations, high absenteeism, and many other factors. Klassen and Chui (2010) believe that mutual satisfaction among members of the educational system is essential for stability.

The literature highlighted the situational and individual factors as the main categories of determinant factors in teachers’ job satisfaction (Gimenez and Tachizawa, 2012). Numerous studies have been conducted to determine various elements that might influence job satisfaction and individuals’ psychological status. Elements such as job characteristics (Judge et al., 2017), working conditions (Puni et al., 2018), workplace atmosphere (Escardíbul and Afcha, 2017), culture (Dwiyanti et al., 2019), stress levels (Dreer, 2021), educational system (Capecchi and Piccolo, 2016), hourly workload (Abate et al., 2018), pay and reward (Berliana et al., 2018), co-workers and students (Dwiyanti et al., 2019), and ambiguity and conflict (Escardíbul and Afcha, 2017) are among the most crucial factors that might have influenced teachers’ job satisfaction.

For instance, Dreer (2021) investigated the relationships between teachers’ well-being in case of engagement, positive emotions, meaning, relationships, achievement, and their job satisfaction. In his study, Dreer (2021) found that there is a strong positive relationship between teachers’ job satisfaction and their well-being. He suggested that among the mentioned factors, positive emotions provided a central role in predicting teachers’ job satisfaction. In another study, Chan et al. (2020) focused on the relationships between self-efficacy and job satisfaction among Chinese teaching assistants. Employing the social cognitive theory of self-efficacy and self-determination theory, they found that teaching assistants’ self-efficacy is positively related to their job satisfaction. Similarly, Lee et al. (2020) investigated the relationships among job satisfaction, emotional intelligence, emotional exhaustion, and subjective well-being in high school athletic directors. They found that there is a statistically significant relationship between job satisfaction and well-being.

While many studies have been conducted on job satisfaction, more research studies are required. Because of the intricacy of the nature of both EFL teachers’ characteristics and teaching environments’ features, it is required to conduct multilevel research studies to determine the interrelations between other factors such as teachers resilience that might influence teachers’ job satisfaction and well-being.

### Teacher Resilience

Another important factor affecting teachers’ well-being is resilience. Resilience as another factor of positive psychology refers to the teachers’ ability to cope with difficult situations (Castro et al., 2010). It is a human ability to adapt effectively to risk factors (Mansfield et al., 2012; Lee, 2017). It is a person’s ability to strike a biological-psychological-spiritual balance in the face of threatening situations and goes beyond surviving the stresses and hardships of life (Merida-Lopez and Extremera, 2017). The level of resilience affects teachers’ ability to manage responses and reactions, which is a difficult and complex process. In this regard, it is mentioned as a factor that can improve the quality and professional standards (Paris, 2013). Resilience is very important in education for three reasons. First, it is effective for the teacher’s expectations of students because teachers are a model for demonstrating resilient behavior (Peters and Pearce, 2012). Second, professional teaching is a difficult and complex process; it requires that a teacher deals with the ambiguities and difficulties encountered in the classroom correctly and logically, and this requires a resilient style of behavior (Judge et al., 2017; Proietti Ergün and Dewaele, 2021). Teachers, on the one hand, have to manage their stress, and on the other hand, they have to perform their professional duties properly in order to maintain their motivation and commitment to the teaching profession in a timely manner (Hoigaard et al., 2012; Mellati et al., 2015; Derakhshan et al., 2019). Third, resilience means the ability to solve problems, quickly retrieve possible solutions, and act boldly in the face of a variety of problems, and it is precisely related to a sense of commitment to work, self-management, and motivation in teaching to achieve students’ all-round achievements (Gimenez and Tachizawa, 2012). Researchers believe that resilience is a process that is under the control of personal characteristics and a specific learning environment (Kangas-Dick and O’Shaughnessy, 2020). Teacher trainers argued that EFL teachers should be equipped with teacher resilience as a prerequisite instrument for language teaching if they want to be successful in their teaching contexts (Mansfield et al., 2016; Ainsworth and Oldfield, 2019).

Several studies focused on teachers’ resilience. For instance, Papatraianou and Le Cornu (2014) argued that individual and professional groups in the teaching environment provide extraordinary opportunities for teachers to support them in their teaching experiences. In another study, Peters and Pearce (2012) stated that school and university leaders’ support enhances teachers’ resilience and their professional development. Similarly, Taxer et al. (2019) believed that positive teacher-learner relationships are a great source of energy for teachers to enhance their resilience and protect themselves against factors such as emotional exhaustion.

One of the pillars of teachers’ well-being is nurturing positive social relationships in the workplace. Some studies examine the basic elements that lead to positive relationships, which are mainly derived from social and emotional competencies (Lee et al., 2020). They provide more information on how to build and develop relationships with language learners from the perspectives of intercultural and interpersonal relationships. They also explore ways to increase positive group dynamics and the value of joint activities (Mills, 2011). Review of the related literature suggested that teachers’ job satisfaction and teachers’ resilience might influence teachers’ well-being (Shoshani and Eldor, 2016; Xie and Derakhshan, 2021). To confirm the findings of the previous studies in the Chinese EFL context, the present study investigated the relationships between Chinese EFL teachers’ job satisfaction, resilience, and their psychological well-being by raising the following research questions.

### Research Questions

(1) Are there any significant relationships between Chinese EFL teachers’ job satisfaction, resilience, and their psychological well-being?
(2) Do Chinese EFL teachers’ job satisfaction and resilience predict their psychological well-being?

## Materials and Methods

### Participants and Research Context

To collect the required data, 343 Chinese EFL teachers with different academic qualifications including both genders (91.84% females, 6.71% males, and 1.46% prefer not to specify), various academic degrees (i.e., 39.36% Bachelor of Arts, 25.95% Diploma, 24.78% Master of Arts, 9.33% Other, and 0.58% Ph.D.) and different majors (i.e., 55.1% English Education, 18.66% English Language and Literature, 15.74% Other, 4.66% Translation, 3.79% Applied Linguistics, and 2.04% Linguistics) participated in the current research study voluntarily. To ensure the generalizability of outcomes, the EFL teachers were chosen purposefully from different cities and provinces of China (i.e., Beijing, Tianjin, Henan Province, Guizhou Province, Guangdong Province, and Hubei Province, etc.), different teaching experiences (*M* = 4) and different age levels (*M* = 38). Among these participants, there are 140 from primary schools, 129 from middle schools, 36 from high schools, 27 from colleges and universities, and 11 from other institutes, and they were selected by using the Wechat phone app through the Questionnaire Star. More demographic information is shown in Table 1.

**TABLE 1:** Participants’ demographic information.

Demographic information	No.
**Gender**	
Male	23
Female	315
Others	0
Prefer not to specify	5
**Age**	
Less than 35	141
36–45	134
46–55	64
Over 55	4
**Major**	
Applied linguistics	13
Linguistics	7
English language literature	64
English language translation	16
English education	189
Other	54
**Last academic degree obtained**	
Diploma	89
Associate of arts	0
Bachelor of arts	135
Master of arts	85
Ph.D.	2
Other	32
**Teaching experience**	
1–5	53
6–10	63
11–15	74
16–20	46
21–25	48
More than 25	59
**Level of education you are currently teaching**	
Primary school	140
Middle school	129
High school	36
University	27
Other	11

### Instrument and Data Collection Procedures

In this study, the Questionnaire Star, an online questionnaire program was adopted to collect data from August 19th to August 31st, 2021. The questionnaire contains 61 questions; 26 questions about job satisfaction, ten questions about teacher resilience, and 25 questions about teacher well-being. And all of these were based on the participants’ willingness. In order to guarantee the trustworthiness of this study, all participants were fully informed of how to fill out the questionnaires and assured that their responses and personal information would be remained confidential and be used exclusively in the current investigation. They were also informed of their rights to free withdrawal from the study at any stage of the study. Then, the collected data were double-checked for possible mistakes before being processed for further statistical analysis. Finally, the probe into the research questions was conducted based on the data.

### Data Analysis

#### Pre-processing of the Data

Before starting to do the analysis, the data went through pre-check processes to exclude the inappropriate data. Primarily, 343 answers were obtained from the administration of the questionnaires. No missing answer was found in the data, and the data was, first, inspected for patterns. Consequently, 21 cases (Case No. 6, 22, 40, 44, 123, 140, 147, 159, 189, 199, 208, 212, 229, 274, 278, 288, 289, 291, 309, 322, and 341) with constant/increasing/decreasing pattern were identified and excluded. Then, the standard deviation of respondents’ answers was calculated and those with values below 0.5 were inspected for unengagement. No such a case was found. Therefore, as a result of data screening, 322 respondents were kept for the main analysis.

#### Construct Validity

Initially, to make sure of the construct validity, a CFA was performed. The initial model had two constructs (Job Satisfaction and Well-being) with items in second order and one (Resilience) with first order. Then, each construct was probed for non-significant loadings in unstandardized estimation and/or low estimates (below 0.5) in standardized estimation. Table 2 shows the results. As reported, no non-significant unstandardized estimates were found. However, three items from job satisfaction, i.e., items 16, 22, and 25, had standardized estimates below 0.45. These items were excluded before going forward.

**TABLE 2:** Unstandardized and standardize estimates of the initial CFA model.

			Unstandardized				Standardized
			Estimate	S.E.	C.R.	*P*	Estimate
S01	<−−−	SF	1.000				0.719
S02	<−−−	SF	1.167	0.088	13.332	0.000	0.792
S03	<−−−	SF	1.016	0.086	11.806	0.000	0.699
S04	<−−−	SF	0.906	0.088	10.280	0.000	0.608
S05	<−−−	SF	1.139	0.089	12.805	0.000	0.759
S06	<−−−	SF	1.199	0.091	13.200	0.000	0.784
S07	<−−−	SF	1.013	0.089	11.421	0.000	0.676
S08	<−−−	WI	1.000				0.624
S09	<−−−	WI	1.121	0.104	10.789	0.000	0.741
S10	<−−−	WI	1.288	0.107	12.053	0.000	0.889
S11	<−−−	WI	1.170	0.104	11.294	0.000	0.791
S12	<−−−	WI	0.938	0.101	9.270	0.000	0.609
S13	<−−−	SI	1.000				0.690
S14	<−−−	SI	1.199	0.116	10.294	0.000	0.787
S15	<−−−	SI	0.748	0.102	7.320	0.000	0.482
S16	<−−−	SI	0.667	0.110	6.069	0.000	0.393
S17	<−−−	SI	0.884	0.102	8.627	0.000	0.581
S18	<−−−	LR	1.000				0.649
S19	<−−−	LR	0.836	0.106	7.878	0.000	0.572
S20	<−−−	LR	0.682	0.101	6.741	0.000	0.470
S21	<−−−	LR	1.078	0.123	8.772	0.000	0.677
S22	<−−−	LR	0.454	0.084	5.391	0.000	0.363
S23	<−−−	CR	1.000				0.715
S24	<−−−	CR	0.586	0.079	7.423	0.000	0.490
S25	<−−−	CR	0.413	0.073	5.660	0.000	0.366
S26	<−−−	CR	1.071	0.107	9.982	0.000	0.780
R01	<−−−	RS	1.000				0.662
R02	<−−−	RS	1.025	0.091	11.300	0.000	0.701
R03	<−−−	RS	1.061	0.095	11.126	0.000	0.688
R04	<−−−	RS	1.174	0.098	11.933	0.000	0.746
R05	<−−−	RS	1.137	0.102	11.152	0.000	0.690
R06	<−−−	RS	1.185	0.094	12.564	0.000	0.793
R07	<−−−	RS	1.138	0.096	11.878	0.000	0.742
R08	<−−−	RS	1.215	0.096	12.677	0.000	0.802
R09	<−−−	RS	1.308	0.105	12.488	0.000	0.788
R10	<−−−	RS	1.204	0.097	12.358	0.000	0.778
W01	<−−−	IFW	1.000				0.666
W06	<−−−	IFW	1.164	0.098	11.859	0.000	0.741
W11	<−−−	IFW	1.027	0.082	12.482	0.000	0.787
W16	<−−−	IFW	1.167	0.092	12.650	0.000	0.799
W21	<−−−	IFW	1.169	0.086	13.513	0.000	0.867
W02	<−−−	TW	1.000				0.740
W07	<−−−	TW	1.060	0.071	14.921	0.000	0.814
W12	<−−−	TW	0.978	0.069	14.097	0.000	0.773
W17	<−−−	TW	1.249	0.075	16.738	0.000	0.903
W22	<−−−	TW	1.182	0.074	16.039	0.000	0.868
W03	<−−−	FCW	1.000				0.692
W08	<−−−	FCW	1.234	0.095	12.994	0.000	0.780
W13	<−−−	FCW	0.978	0.081	12.135	0.000	0.725
W18	<−−−	FCW	1.165	0.088	13.210	0.000	0.794
W23	<−−−	FCW	1.140	0.092	12.431	0.000	0.743
W04	<−−−	PRW	1.000				0.653
W09	<−−−	PRW	0.759	0.076	10.024	0.000	0.628
W14	<−−−	PRW	0.970	0.080	12.076	0.000	0.786
W19	<−−−	PRW	0.928	0.080	11.561	0.000	0.744
W24	<−−−	PRW	0.776	0.078	9.975	0.000	0.625
W05	<−−−	DIW	1.000				0.722
W10	<−−−	DIW	0.711	0.079	9.021	0.000	0.519
W15	<−−−	DIW	1.169	0.083	14.095	0.000	0.804
W20	<−−−	DIW	1.028	0.073	14.049	0.000	0.801
W25	<−−−	DIW	1.006	0.095	10.636	0.000	0.611

**FIGURE 1:**
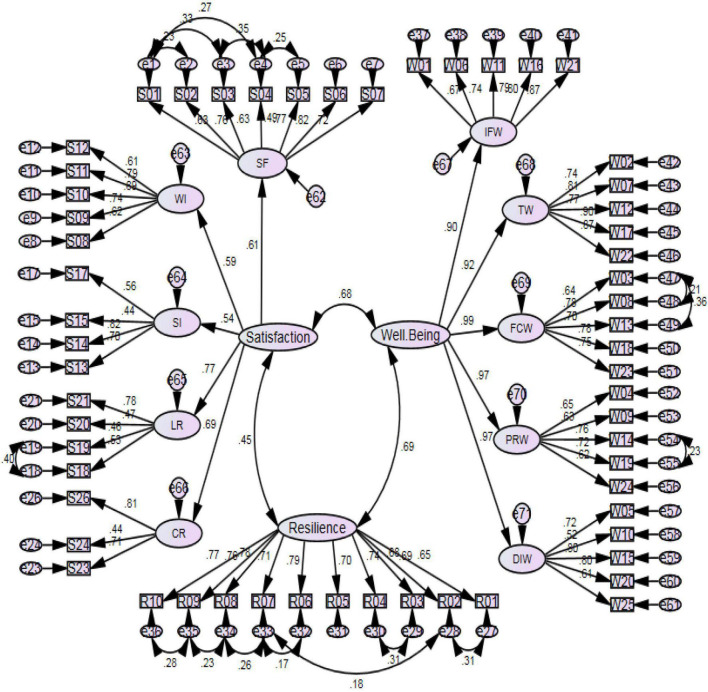
The final modified CFA model with standardized estimates.

Next, the modification indices with the threshold of 10 were checked and the suggestions that were not contradictory to the literature were applied. Figure 1 delineates the final modified CFA model.

Table 3 presents the descriptive statistics for the component of the model after regression imputation. Regression imputation works like calculation of the average scores for each component; yet it is a more accurate measure as it takes into account the weighted share of each item in calculating the average. In other words, each item is weighted based on its share of explaining the average variance of its component.

**TABLE 3:** Descriptive statistics of the scores after regression imputation.

	*N*	Minimum	Maximum	Mean	SD	Skewness	Kurtosis
SF	322	0.39	3.78	1.4286	0.79039	0.705	–0.161
WI	322	0.78	4.77	2.5361	0.99046	0.204	–0.795
SI	322	0.27	4.41	1.6107	0.84744	0.634	0.006
LR	322	0.14	3.64	1.7568	0.74418	0.188	–0.700
CR	322	0.23	4.57	2.7865	0.94079	–0.200	–0.706
Satisfaction	322	0.37	3.85	2.0940	0.63433	0.068	–0.462
Resilience	322	0.11	3.28	2.2949	0.59927	–0.386	–0.105
IFW	322	1.00	4.26	3.2828	0.73155	–0.773	0.026
TW	322	0.56	4.64	3.3297	0.93181	–0.641	–0.311
FCW	322	0.97	4.03	3.0330	0.68863	–0.665	–0.168
PRW	322	1.26	5.08	3.8240	0.85043	–0.655	–0.235
DIW	322	1.10	4.83	3.6046	0.84026	–0.618	–0.297
Well-Being	322	0.97	3.94	2.9480	0.67111	–0.635	–0.248

As reported in Table 3, all distributions of the scores that showed normalcy as both skewness and kurtosis values were below the absolute value of 2.

Next, the composite reliability (CR) and discriminant validity for each factor was examined (Table 4). As reported, all of the variables had CR values above 0.7, which reveals acceptable reliability. Moreover, the squares root of average variance extracted (AVE) (the bold values in the table) was above inter-correlations of the factors, indicating discriminant validity, according to Fornell and Larcker (1981).

**TABLE 4:** Composite reliability and discriminant validity of the factors.

		Fornell-Larcker criterion		
	C.R.	Satisfaction	Resilience	Well-Being
Satisfaction	0.843	**0.682**		
Resilience	0.919	0.450**	**0.729**	
Well-Being	0.979	0.676**	0.691**	**0.950**

**Correlation is significant at 0.01.

The inspection of the correlations (values not in bold under Fornell-Larcker Criterion) documented that there are significant correlations between all pairs of factors. Strong correlations were found between satisfaction and well-being (*r* = 0.676) as well as resilience and well-being (*r* = 0.691) while the correlation between satisfaction and resilience was moderate (*r* = 0.45). Moreover, the root of AVE for each factor (the bold values in the table), was safely above the correlation of that variable with others, indicating discriminant validity (Fornell and Larcker, 1981).

As the results of correlation analysis, above, showed well-being has a strong correlation with both job satisfaction and resilience, a prediction model was created to measure the predictability of well-being by the two other variables. Table 5 reports the results of the analysis. It should be noted that, in running the analysis, the imputed values extracted from CFA were used.

**TABLE 5:** Results of multiple linear regression analysis with SEM.

			Weight	S.E.	C.R.	*P*	β	*R* ^2^	Multiple correlation *R*^2^
Well-Being	<−−−	Satisfaction	0.575	0.035	16.361	0.000	0.544	0.296	0.564
Well-Being	<−−−	Resilience	0.493	0.037	13.250	0.000	0.440	0.194	
Satisfaction	<−−>	Resilience	0.207	0.024	8.585	0.000	0.546		

**FIGURE 2:**
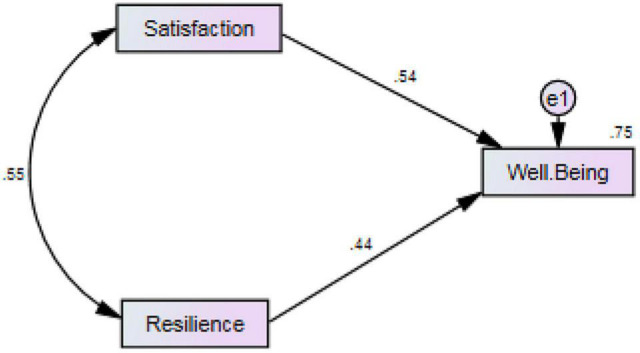
The final measurement model.

As reported in Table 5, after taking into account the covariance between job satisfaction and resilience, the two variables could jointly predict 56.4 of the variance in psychological well-being. Both variables were the significant predictor of well-being, while satisfaction was better predictor (β = 0.544, *p* = 0.000 < 0.01), uniquely explaining 29.6 of well-being’s variance. Resilience was also proved to be a significant predictor of well-being (β = 0.44, *p* = 0.000 < 0.01), uniquely explaining 19.4 of its variance. The prediction model is depicted in Figure 2, below.

## Discussion

This particular study was to investigate the relationship among job satisfaction, the resilience of Chinese EFL teachers and their well-being. The findings of correlation analysis showed that there was a significant relationship among teachers’ job satisfaction, resilience, and their well-being. First, the findings revealed that teachers’ resilience operated as a defensive factor for their well-being. Second, the findings also indicated that the variable, job satisfaction, was positively associated with teachers’ resilience and their well-being (Wang et al., 2021). Therefore, to develop Chinese EFL teachers’ well-being, it is required to promote their resilience and job satisfaction.

These findings are consistent with the existing literature on these notions; studies confirmed that teachers with a high level of satisfaction and resilience are better able to establish positive relationships, resulting in higher levels of teachers’ well-being (Greenier et al., 2021). Similar to Mousavi et al. (2012), the findings of the study confirmed that job satisfaction and resilience increase teachers’ well-being and ability to do things and make them more resilient to job stress. The findings confirmed what Dreer (2021) found that there is a strong relationship between teachers’ job satisfaction and their well-being.

Feeling satisfied with the work environment can strengthen positive relationships in the workplace, which in turn increases confidence and the development of skills to deal with potential problems in educational settings (Fathi et al., 2021). When teachers enjoy their work, their personal lives outside the educational environment improve, and conversely, a dissatisfied teacher transmits his or her negative attitude to the educational environment and society. Due to the adverse effects of job dissatisfaction, the benefits of teachers’ job satisfaction will affect all people in educational settings as well as society (Bowling et al., 2010; Beltman et al., 2011; Capecchi and Piccolo, 2016).

Psychologists believe that job satisfaction is directly related to the human psyche and, of course, a person’s productivity. If teachers feel satisfied in the work they do, it will cause them to flourish and increase their productivity, and there will be no sign of fatigue and sluggishness in their work (Dwiyanti et al., 2019). Also, if teachers have job dissatisfaction, depression and boredom affecting all aspects of their lives, they cannot have a good return to their work. Therefore, in order to increase the level of productivity, administrators should use their educational environment to create a sense of satisfaction in teachers and remove its obstacles.

Another important factor in Chinese EFL teachers’ well-being is their resilience. The findings of the study indicated that there is a positive and direct relationship between teachers’ high level of resilience and their well-being (Friedman and Kern, 2014). Researchers believe that resilience is essentially the process of successfully adapting to life’s challenges or experiences. Resilient and flexible teachers overcome problems, recover from failures, and can progress under intense and constant pressure without taking action through harmful and inefficient methods. After recovering from their traumatic experiences, most of these teachers perform stronger, better, and wiser.

In other words, resilient teachers maintain a high level of motivation and achievement despite stressful events and situations. Therefore, the role of motivation may be central to teachers’ resilience. Schools and educational institutions can help vulnerable teachers succeed through strategies such as strategic planning and improving school-level performance. Creating such a situation helps teachers to be more stable, focused, and feels better about themselves, all of which will affect their performance as well as students’ learning and academic achievement (Gu and Day, 2007). There are also other long-term benefits. Some of the processes and methods involved in forming resilience allow teachers to access opportunities they have never had before. For example, skills training and internships provide long-term benefits for teachers, including better physical health, increased life expectancy, and long-term mental health, by increasing teachers’ resilience levels.

The findings showed that resilience is a multidimensional concept and includes cognitive, emotional, social, behavioral, and psychological/physical dimensions. Teacher resilience is not only a scientific-technical concept, but also a kind of being combined with calmness and culture, and behavior, which is considered for teachers as an intellectual and behavioral model. On the one hand, resilience shows knowledge, thoughts, and behavior of a teacher in different situations, especially those that are accompanied by fatigue and anxiety. This creates a firm, calm and effective behavior in his or her personality in learning environments. Students and teachers, on the other hand, interact with each other to understand the influential spiritual and moral capacities of resilience in educational settings. There is a difference between teachers who make hasty and emotional decisions and those who can manage situations, crises, and emergencies wisely, calmly, and masterfully.

## Conclusion and Pedagogical Implications

The findings of the current study demonstrated that job satisfaction, resilience, and well-being as main factors of positive psychology are complex multidimensional concepts. Since the factors that influence them and their constructive implications are significantly intertwined with each other, it is necessary to consider them as an integrated structure to create a positive effect. Considering the results of the present study and the findings of other studies, the following suggestions are presented.

Teachers’ resilience and job satisfaction as two important notions in the language teaching field of study should be further studied in similar or dissimilar educational contexts and EFL teachers should be more effectively cultured and educated through human resource development systems in which administrators can play a vital role.

It is recommended to pay attention to strengthening the cognitive dimension of resilience and job satisfaction through holding seminars and classes in educational environments. Raising resilience and job satisfaction issues in meetings of EFL teachers’ councils and principals is strongly recommended to emphasize resilience and job satisfaction literature among EFL teachers. The findings of study confirmed the culture and context dependence of positive psychology and its components in language teaching; therefore, teaching culture and context should be considered in designing EFL teachers’ education programs.

The emotional dimension of resilience and job satisfaction requires attention to the emotional-psychological aspects of EFL teachers and their greater self-improvement, which is recommended to improve through in-service courses in which the teacher is encouraged and involved to strengthen the ability of emotional-psychological control. It is also recommended to acquaint EFL teachers with emotional resilience and job satisfaction strategies in the form of brochures, competitions and action research.

Due to the culture and context dependence of these notions, the findings of the present study should be confirmed in other EFL contexts. Future studies are required to shed light on latent aspects of positive psychology in different EFL contexts.

## Data Availability Statement

The raw data supporting the conclusions of this article will be made available by the authors, without undue reservation.

## Ethics Statement

The studies involving human participants were reviewed and approved by Academic and Ethics Committee of Henan University. The patients/participants provided their written informed consent to participate in this study.

## Author Contributions

WH: conceptualized, designed and drafted the manuscript, and submitted to the final version.

## Conflict of Interest

The author declares that the research was conducted in the absence of any commercial or financial relationships that could be construed as a potential conflict of interest.

## Publisher’s Note

All claims expressed in this article are solely those of the authors and do not necessarily represent those of their affiliated organizations, or those of the publisher, the editors and the reviewers. Any product that may be evaluated in this article, or claim that may be made by its manufacturer, is not guaranteed or endorsed by the publisher.
